# Supplementary material for: The Bugs in the Bags: The Risk Associated with the Introduction of Small Quantities of Fruit and Plants by Airline Passengers
Source: Insects. 2022 Jul 10;13(7):617. doi: 10.3390/insects13070617 (PMC9323091; doi:10.3390/insects13070617)
Supplement: Supplementary file 1 [file insects-13-00617-s001.zip › File S1.pdf]

File S1. Summary of the actual preventive actions against the introduction of new pests from Third Countries established by EU Plant Health Regulation Framework

EU-R (Regulation) 2016/2031 [1] requires that all plants and parts of plants intended for trade need to be accompanied by a phytosanitary certificate to enter the EU, unless they are exempted and listed in Annex XI, Part C, of EU-IR (Implementing Regulation) 2019/2072 [2] and EU-IR 2021/2285 [3]. The certificate should guarantee that the cargo is in good phytosanitary status and does not present any potentially dangerous organisms.

- EU-IR 2018/2019 [4] defines the procedures to be followed to perform the risk assessment of high-risk plants, plant products, and other objects within the meaning of Article 42(1) of EU-R 2016/2031 [1].

- EU-IR 2021/632 [5] lays down rules for the application of EU-R 2017/625 [6] and concerns the lists of animals and products to be subject to controls at border inspection posts, with the exceptions reported in the EU-CDR (Commission Delegated Regulation) 2019/2122 [7].

- to reduce the possibility of pest introduction with international trade, wood materials are regulated under specific phytosanitary measure identified by the IPPC and is the basis of the ISPM-15 (International Standards for Phytosanitary Measures) [8] that establishes the heat treatment and marking for raw wood packaging [9].

Essential bibliography (reported in the main text also)

1. EU. Consolidated text: Regulation (EU) 2016/2031 of the European Parliament of the Council of 26 October 2016 on protective measures against pests of plants amending Regulations (EU) No 228/2013, (EU) No 652/2014 and (EU) No 1143/2014 of the European Parliament and of the Council and repealing Council Directives 69/464/EEC, 74/647/EEC, 93/85/EEC, 98/57/EC, 2000/29/EC, 2006/91/EC and 2007/33/EC. **2019**. Available online: http://data.europa.eu/eli/reg/2016/2031/2019-12-14 (accessed on 23 May 2022)
2. EU. Consolidated text: Commission Implementing Regulation (EU) 2019/2072 of 28 November 2019 establishing uniform conditions for the implementation of Regulation (EU) 2016/2031 of the European Parliament and the Council, as regards protective measures against pests of plants, and repealing Commission Regulation (EC) No 690/2008 and amending Commission Implementing Regulation (EU) 2018/2019. **2022**. Available online: https://eur-lex.europa.eu/legal-content/EN/TXT/?uri=CELEX%3A02019R2072-20220411 (accessed on 23 May 2022)
3. EU. Commission Implementing Regulation (EU) 2021/2285 of 14 December 2021 amending Implementing Regulation (EU) 2019/2072 as regards the listing of pests, prohibitions and requirements for the introduction into, and movement within, the Union of plants, plant products and other objects, and repealing Decisions 98/109/EC and 2002/757/EC and Implementing Regulations (EU) 2020/885 and (EU) 2020/1292 2020/1292. **2021**. Available online: https://eur-lex.europa.eu/eli/reg_impl/2021/2285/oj (accessed on 23 May 2022)
4. EU. Consolidated text: Commission Implementing Regulation (EU) 2018/2019 of 18 December 2018 establishing a provisional list of high risk plants, plant products or other objects, within the meaning of Article 42 of Regulation (EU) 2016/2031 and a list of plants for which phytosanitary certificates are not required for introduction into the Union, within the meaning of Article 73 of that Regulation. **2022**. Available online: https://eur-lex.europa.eu/legal-content/EN/TXT/?uri=CELEX%3A02018R2019-20220331 (accessed on 23 May 2022)
5. EU. Consolidated text: Commission Implementing Regulation (EU) 2021/632 of 13 April 2021 laying down rules for the application of Regulation (EU) 2017/625 of the European Parliament and of the Council as regards the lists of animals, products of animal origin, germinal products, animal by-products and derived products, composite products, and hay and straw subject to official controls at border control posts, and repealing Commission Implementing Regulation (EU) 2019/2007 and Commission Decision 2007/275/EC. **2022**. Available online: http://data.europa.eu/eli/reg_impl/2021/632/2022-03-02 (accessed on 23 May 2022)
6. EU. Consolidated text: Regulation (EU) 2017/625 of the European Parliament and of the Council of 15 March 2017 on official controls and other official activities performed to ensure the application of food and feed law, rules on animal health and welfare, plant health and plant protection products, amending Regulations (EC) No 999/2001, (EC) No 396/2005, (EC) No 1069/2009, (EC) No 1107/2009, (EU) No 1151/2012, (EU) No 652/2014, (EU) 2016/429 and (EU) 2016/2031 of the European Parliament and of the Council, Council Regulations (EC) No 1/2005 and (EC) No 1099/2009 and Council Directives 98/58/EC, 1999/74/EC, 2007/43/EC, 2008/119/EC and 2008/120/EC, and repealing Regulations (EC) No 854/2004 and (EC) No 882/2004 of the European Parliament and of the Council, Council Directives 89/608/EEC, 89/662/EEC, 90/425/EEC, 91/496/EEC, 96/23/EC, 96/93/EC and 97/78/EC and Council Decision 92/438/EEC (Official Controls Regulation). **2022**. Available online: http://data.europa.eu/eli/reg/2017/625/2022-01-28 (accessed on 23 May 2022).
7. EU. Consolidated text: Commission Delegated Regulation (EU) 2019/2122 of 10 October 2019 supplementing Regulation (EU) 2017/625 of the European Parliament and of the Council as regards certain categories of animals and goods exempted from official controls at border control posts, specific controls on passengers' personal luggage and on small consignments of goods sent to natural persons which are not intended to be placed on the market and amending Commission Regulation (EU) No 142/2011. **2022**. Available online: http://data.europa.eu/eli/reg_del/2019/2122/2021-12-20 (accessed on 23 May 2022).

8. Food and Agriculture Organization of the United Nations (FAO). International Standards for Phytosanitary Measures (ISPM) No. 15 Regulation of wood packaging material in international trade. 2018. Available online: https://www.fao.org/3/mb160e/mb160e.pdf (accessed on 23 May 2022).
9. Allen, E.; Noseworthy, M.; Ormsby, M. Phytosanitary measures to reduce the movement of forest pests with the international trade of wood products. *Biol. Invasions* **2017**, *19*, 3365–3376.
