# Supplementary material for: The Bugs in the Bags: The Risk Associated with the Introduction of Small Quantities of Fruit and Plants by Airline Passengers
Source: Insects. 2022 Jul 10;13(7):617. doi: 10.3390/insects13070617 (PMC9323091; doi:10.3390/insects13070617)
Supplement: Supplementary file 1 [file insects-13-00617-s001.zip › Table S1.pdf]

Table S1. Plant material species seized at BCPs of Campania region and number of loads resulted positive at the presence of arthropods. AP, airport; PN, port of Naples; PS port of Salerno.

Botanical species	n. of entry	BCP	Positive	Botanical species	n. of entry	BCP	Positive
Abelmoschus esculentus	3	AP	1	*Loropetalum chinense*	2	PN	0
Aegle marmelos	2	AP	0	*Malus domestica*	1	AP	1
Allium cepa	3	AP	0	*Mammillaria elongata*	2	PN	0
Ananas comosus	1	AP	0	*Mammillaria hahniana*	1	PN	0
Annona cherimola	1	AP	0	*Mammillaria* sp.	1	PN	0
Annona glabra	1	AP	0	*Mammillaria spinosissima*	1	PN	0
Areca catechu	1	AP	0	*Mangifera indica*	20	AP	2
Artocarpus heterophyllus	2	AP	1	*Manilkara zapota*	1	AP	0
Asplenium nidus	1	AP	0	*Mentha* sp.	1	AP	0
Baccaurea ramiflora	2	AP	0	*Momordica charantia*	3	AP	1
Bambuseae	1	AP	0	*Moringa oleifera*	2	AP	0
Beaucarnea	1	PN	1	*Musa acuminata*	2	AP	0
Bougainvillea glabra	1	PN	0	*Musa paradisiaca*	5	AP	0
Bougainvillea spectabilis	1	PN	0	*Nigella sativa*	1	AP	0
Butia sp.	1	PN	0	*Olea europaea*	1	AP	0
Calocasia esculenta	1	AP	0	*Oryza sativa*	1	PN	1
Capsicum sp.	11	AP	0	*Parodia schumanniana*	1	PN	0
Carica papaya	8	AP	0	*Passiflora edulis*	3	AP	0
Ceiba sp.	1	PN	1	*Passiflora* sp.	2	AP	0
Cereus hildmannianus	2	PN	0	*Persea americana*	11	AP	0
Chorisia sp.	1	PN	0	*Phyllantus emblica*	1	AP	0
Cinnamomum verum	1	AP	0	*Phyllostachys nigra*	1	PN	0
Citrus × bergamia	1	AP	0	*Phyllostachys pubescens*	1	PN	0
Citrus aurantiifolia	4	AP	0	*Physalis peruviana*	1	AP	0
Citrus limon	6	PN	4	*Pinus parviflora*	1	PN	1
Citrus limon	1	PS	0	*Piper betle*	12	AP	0
Citrus maxima	1	AP	0	*Pisum sativum*	1	AP	0
Citrus paradisi	2	AP	0	*Podocarpus macrophyllus*	1	PN	1
Citrus sp.	13	AP	0	*Psidium guajava*	26	AP	3
Citrus aurantium	6	PN	4	*Punica granatum*	3	AP	0
Cocos nucifera	2	AP	0	*Saccharum officinarum*	4	AP	0
Cynara scolymus	1	AP	0	*Sanseveria trifascata*	3	PN	0
Cyperus papyrus	1	AP	0	*Solanum aethiopicum*	5	AP	3
Diospyros kaki	1	AP	0	*Solanum betaceum*	2	AP	0
Dioscorea spp.	2	AP	2	*Solanum torvum*	2	AP	0
Dioscorea spp.	1	PS	0	*Solanum tuberosum*	1	PS	0
Echinocactus grusonii	7	PN	0	*Solanum tuberosum*	1	AP	0
Euphorbia tirucalli	2	PN	0	*Spondias dulcis*	3	AP	0
Fernaldia pandurata	1	AP	0	*Tamarindus indica*	1	AP	1
Ficus hispida	1	AP	0	*Terminalia catappa*	1	AP	1
Ficus microcarpa	1	PS	1	*Theobroma cacao*	1	AP	1
Ficus microcarpa	9	PN	2	*Trichosanthes cucumerina*	1	AP	1
Garcinia mangostana	1	AP	0	*Triplochiton scleroxylon*	1	PN	1
Gymnocalycium baldianum	1	PN	0	*Viburnum tinus*	1	AP	0
Ilex crenata	1	PN	0	*Xhantosoma* spp.	1	AP	0

Jubaea chilensis	1	PN	0	*Zelkova* sp.	2	PN	0
Lablab purpureus	2	AP	1	*Zingiber officinale*	2	AP	0
Lagerstroemia indica	1	PN	0	*Ziziphus jujuba*	14	AP	1
Lansium parasiticum	1	AP	1	*Ziziphus mauritiana*	2	AP	0
Limonia acidissima	1	AP	0				
