# Supplementary figures and images for: The Bugs in the Bags: The Risk Associated with the Introduction of Small Quantities of Fruit and Plants by Airline Passengers
Source: Insects. 2022 Jul 10;13(7):617. doi: 10.3390/insects13070617 (PMC9323091; doi:10.3390/insects13070617)

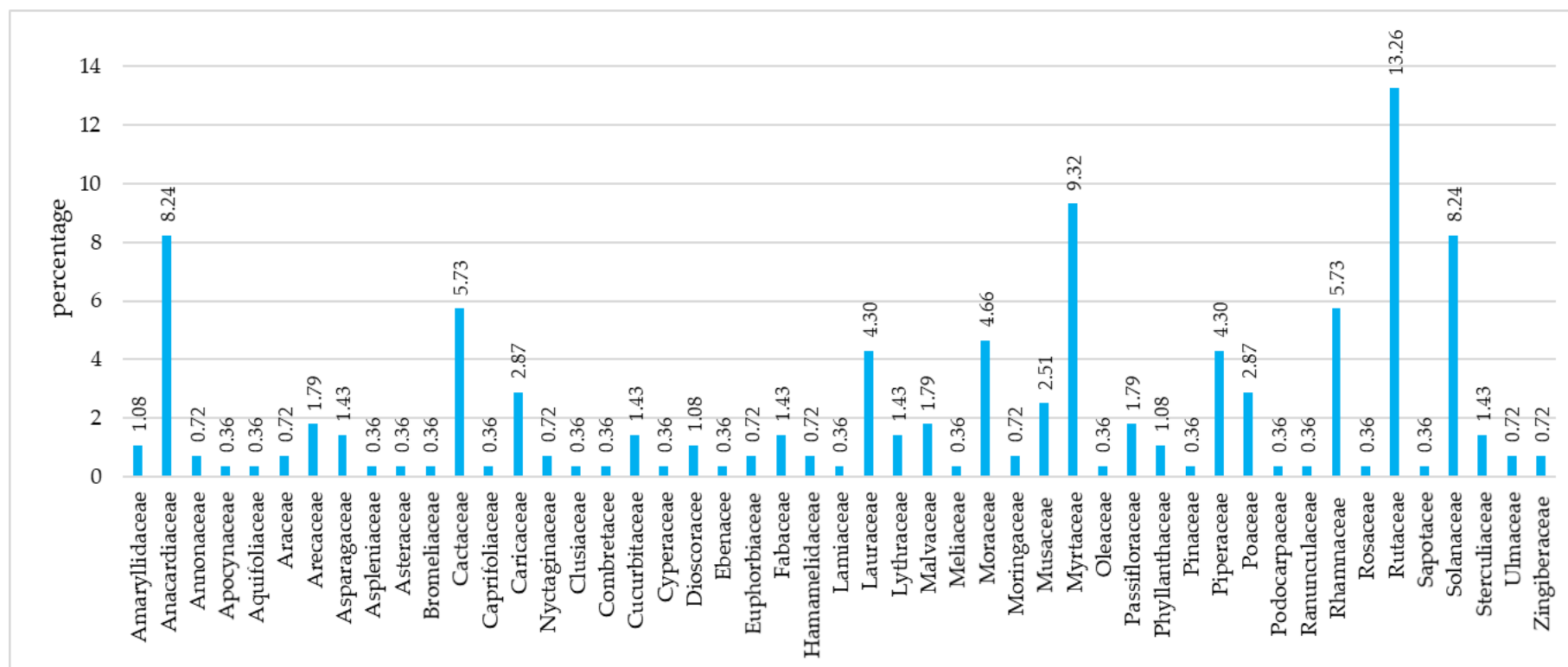

**Figure S1.** Percentage of botanical families which seized plant material belong.

Supplement: Supplementary file 1 [file insects-13-00617-s001.zip › Figure S1.pdf]
